# Clinical activity in general practice before sarcoma diagnosis: an Australian cohort study

**DOI:** 10.3399/BJGP.2023.0610

**Published:** 2024-06-25

**Authors:** Meena Rafiq, Jasper de Boer, Jasmine Mar, Jayesh Desai, Susie Bae, David E Gyorki, Claudia Di Bella, Georgios Lyratzopoulos, Jeremy H Lewin, Jon Emery

**Affiliations:** Department of General Practice and Centre for Cancer Research, University of Melbourne, Melbourne, Australia, and Epidemiology of Cancer Healthcare and Outcomes (ECHO) Group, Department of Behavioural Science and Health, Institute of Epidemiology and Health Care (IECH), UCL, London, UK.; Victorian Comprehensive Cancer Centre (VCCC), VCCC Alliance, and Australian and New Zealand Children’s Haematology/Oncology Group (ANZCHOG), Melbourne, Australia.; Peter MacCallum Cancer Centre, and Australian and New Zealand Sarcoma Association (ANZSA), Melbourne, Australia.; VCCC Alliance, and Peter MacCallum Cancer Centre, and Sir Peter MacCallum Department of Oncology, University of Melbourne, Melbourne, Australia.; Peter MacCallum Cancer Centre, and Sir Peter MacCallum Department of Oncology, University of Melbourne, Melbourne, Australia.; Peter MacCallum Cancer Centre, and Sir Peter MacCallum Department of Oncology, University of Melbourne, Melbourne, Australia.; St Vincent’s Private Hospital, Melbourne, Australia.; ECHO Group, Department of Behavioural Science and Health, IECH, UCL, London, UK.; VCCC Alliance, and Peter MacCallum Cancer Centre, and ANZSA, and Victorian AYA Cancer Service, Melbourne, Australia.; Department of General Practice and Centre for Cancer Research, University of Melbourne, Melbourne, Australia.

**Keywords:** diagnostic activity, diagnostic time window, general practice, imaging, referral and consultation, sarcoma

## Abstract

**Background:**

Increased time to diagnosis in sarcoma is associated with poor prognosis and patient outcomes. Research is needed to identify whether opportunities to expedite the diagnosis of sarcoma in general practice exist.

**Aim:**

To examine pre-diagnostic GP clinical activity before sarcoma diagnosis.

**Design and setting:**

An Australian retrospective cohort study using hospital registry data (Australian Comprehensive Cancer Outcomes and Research Database [ACCORD]) linked to two primary care datasets (Patron and MedicineInsight).

**Method:**

The frequency of general practice healthcare utilisation events (general practice attendances, prescriptions, blood test, and imaging requests) were compared in 377 patients with soft tissue sarcoma (STS) and 64 patients with bone sarcoma (BS) in the year pre-diagnosis. Poisson regression models were used to calculate monthly incidence rate ratios (IRR) for the 24 months pre-diagnosis and estimate inflection points for when healthcare use started to increase from baseline.

**Results:**

In the 6 months pre-diagnosis, patients with sarcoma had a median of 3–4 general practice attendances, around one-third had a GP imaging request (33% [*n* = 21] BS and 36% [*n* = 134] STS), and approximately one in five had multiple imaging requests (19% [*n* = 12] BS and 21% [*n* = 80] STS). GP imaging requests progressively increased up to eight-fold from 6 months before sarcoma diagnosis (IRR 8.43, 95% confidence interval [CI] = 3.92 to 18.15, *P*<0.001) and general practice attendances increased from 3 months pre-diagnosis.

**Conclusion:**

Patients with sarcoma have increased GP clinical activity from 6 months pre-diagnosis, indicating a diagnostic window where potential opportunities exist for earlier diagnosis. Interventions to help identify patients and promote appropriate use of imaging and direct specialist centre referrals could improve earlier diagnosis and patient outcomes.

## Introduction

Each year in Australia approximately 2500 new cases of sarcoma are diagnosed.^1^ Sarcoma, although rare in adults, represents one of the most common forms of cancer among children and adolescents and young adults (AYA), accounting for about 10%–20% of cancers within these groups.^2^ Despite significant advances in the diagnosis and treatment of cancer broadly, outcomes in sarcoma remain disconcertingly low, with nearly 40% of patients dying within 5 years of diagnosis^1^ and poorer outcomes for those with high-grade or metastatic disease.^3^^,^^4^ The poor prognosis is partially attributed to the inherent challenges in diagnosing sarcoma, notably owing to its rarity (represents <1% of all adult malignancies) and heterogeneous symptoms.^5^^,^^6^ As a result, many patients experience lengthy intervals between initial presentation and diagnosis.^7^^–^^10^ This is particularly relevant in AYA, in whom the incidence of cancer is less frequent, placing it low on the list of differential diagnoses when new symptoms arise.^6^^,^^11^ Delays in sarcoma diagnosis are associated with worse quality of life, poorer patient experience,^12^^,^^13^ and larger tumour size at diagnosis,^14^ which is an important prognostic factor in sarcoma.^15^ Therefore, efforts to expedite the diagnosis of sarcoma are essential to improve patient outcomes and experience.

Almost all patients with sarcoma are diagnosed after symptomatic presentation to their GP.^8^^,^^16^ Further research is needed to determine if there are opportunities to expedite the diagnosis of sarcoma in the primary care setting. UK studies have demonstrated that patients with sarcoma often have multiple GP consultations before they are referred to a specialist.^12^^,^^17^ Notably, patients with bone sarcoma (BS) experience one of the highest number of GP consultations pre-diagnosis among all rare cancer types.^17^ It remains to be determined if similar pre-diagnostic increases in GP healthcare use occur in the Australian setting, where the healthcare structure, patient demographics, and clinician and/or patient behaviours differ. In particular, GPs in Australia have direct access to a wide range of specialist investigations, few barriers to rapid investigation, referral autonomy (with the option of informal specialist referrals and formal managed referral pathways), and free movement of patients between public and private health systems.^18^ This increased flexibility could present more or earlier opportunities for expediting diagnosis.

This study aimed to examine trends in various GP clinical activities over time preceding a sarcoma diagnosis in the Australian context. This will help to identify when patients with as-yet-undetected sarcoma first start using primary health care more frequently, how far diagnosis could potentially be brought forward, and where opportunities might exist within primary care to accelerate the diagnostic process.

**Table:** How this fits in

Sarcoma is challenging to diagnose with delays associated with poor patient outcomes and experiences. This study has shown that patients with sarcoma often have multiple GP visits and imaging requests in the year before their diagnosis. Clinical activity in general practice increases from 6 months before sarcoma diagnosis, primarily in the form of imaging requests, indicating that opportunities for a timelier diagnosis may exist in some patients. Primary care interventions to increase awareness of sarcoma symptoms and streamline diagnostic pathways, including promoting and clarifying guidelines to optimise the use of appropriate imaging and direct specialist centre referrals, could improve earlier diagnosis and patient outcomes.

## Method

### Data sources

A retrospective cohort study was conducted using hospital cancer registry data from the Australian Comprehensive Cancer Outcomes and Research Database (ACCORD), linked to two primary care databases,^19^ Patron^20^ and MedicineInsight^21^ (Supplementary Figure S1). The ACCORD sarcoma dataset used in this study contains clinician-recorded data on patients with sarcoma who were seen at the Peter MacCallum Cancer Centre (PMCC) between 2009 and 2021, which is a tertiary referral service managing most patients with sarcoma within the state of Victoria. This includes data from patients with sarcoma diagnosed before 2009 who received care at PMCC after 2009. Patron and MedicineInsight are primary care electronic health record databases containing de-identified information from GP encounters. MedicineInsight covers a representative, nationwide sample of approximately 8% of general practices in Australia,^21^ with a subset of practices located in Victoria used in this study. A second source of general practice data came from the Patron primary care dataset, which contains data from >130 GP clinics in Victoria.^22^

### Study population

All patients with a new soft tissue sarcoma (STS) or BS, diagnosed in ACCORD between 1 January 2002 and 31 July 2021, were identified and the earliest histologically confirmed diagnosis date selected. Patients who did not have linkage to at least one general practice dataset were excluded. In Australia, patients are not required to be registered at a single general practice and can choose to receive care from multiple general practices. As a result, individuals are often simultaneously registered to multiple practices, where they have consulted at some point in their life. To ensure only patients ‘actively’ registered with a linked general practice were included, patients were excluded who did not have at least one GP encounter in the year preceding sarcoma diagnosis.

This criterion was selected as 90% of patients with sarcoma will first present in primary care^8^^,^^16^ and the vast majority of these presentations will be within the 12 months pre-diagnosis,^8^ so this approach should have captured almost all patients with sarcoma active in a linked practice. ACCORD data were extracted on referral route (GP or other), symptoms at the first hospital consultation (mass, pain, systemic symptoms), tumour characteristics (location, behaviour, grade, stage, depth, size), and occurrence of non-diagnostic biopsies pre-diagnosis.

### Defining the exposures

Primary care data were extracted on patient demographics (age at diagnosis and sex) and instances of the following four types of GP clinical events: GP visits; radiological imaging requests; GP-issued prescriptions (for any medication); and blood test requests. Imaging requests were included both as a composite measure, encompassing all X-ray, ultrasound scan (USS), computed tomography (CT), magnetic resonance imaging (MRI), and bone density scan (dual energy X-ray absorptiometry [DXA]) tests, and considering each modality individually. If patients were ‘actively’ registered (at least one encounter a year) at a linked general practice for ≥2 years pre-diagnosis, clinical activity data were extracted from the 24 months pre-diagnosis. If patients were only ‘actively’ registered in the year pre-diagnosis, data were extracted from the 12 months pre-diagnosis.

### Statistical analysis

Sensitivity analyses were conducted to compare patient and tumour characteristics between patients with sarcoma in the final study cohort with all patients with sarcoma in the ACCORD sarcoma registry, and to compare patients with sarcoma with and without linkage to a primary care dataset to ensure they were comparable. In the final study cohort, χ^2^ analyses were then used to compare baseline and tumour characteristics of patients with STS with BS, and the proportion of patients receiving each type of general practice event in the 6 and 12 months preceding sarcoma diagnosis. For each type of GP clinical activity, the proportion of patients with STS and BS having the event in the 6- and 12-month periods pre-diagnosis was initially compared using a binary (any or none) classification, followed by comparison of the total number and average number of events (mean [standard deviation and range] and median [interquartile range]). GP imaging requests were initially analysed using the composite measure, before repeating the analyses for each of the five imaging modalities separately.

Poisson regression models were used to examine trends in primary healthcare use and different types of clinical activity over time. For the four types of clinical events, monthly incidence rates (IRs) were estimated and plotted for each of the 24 months before sarcoma diagnosis. Monthly rate ratios were then calculated, comparing monthly IRs to the baseline rate of each clinical activity in the study population at 24 months before diagnosis. Using previously described methods,^23^^,^^24^ the inflection point, where the rate of each type of clinical activity first starts to increase from the baseline rate (at 24 months before diagnosis), was statistically estimated. This method involved conducting a series of Poisson regression models, using different sequential monthly inflection points, and using the maximum likelihood method to select the model with the highest log likelihood and therefore best fit for the data. Bootstrapping was used to provide confidence intervals (CIs) for each inflection point and the earliest inflection point selected to define the diagnostic window. To take into account any trends in activity before the inflection point, incidence rate ratios (IRRs) were calculated comparing the periods pre- and post- the inflection point or diagnostic window.

To assess the extent of different types of primary care clinical activity among patients with sarcoma over time, the monthly (incident and cumulative) percentage of patients experiencing each type of event was calculated and plotted for the 24 months pre-diagnosis.

## Results

### Patient characteristics

From the 3741 patients with sarcoma in the ACCORD dataset, 1250 (33%) were linked to primary care datasets and 441 (12%) had at least one recorded encounter in a linked general practice in the year before diagnosis. Of the 809 linked patients who were excluded: 200 only had a linked GP encounter after their sarcoma diagnosis, 436 only had historic linked GP encounters (>1 year before diagnosis), 53 patients had both, and 120 had no linked GP encounter recorded between 1980 and 2020. Sensitivity analyses showed that no substantial differences were found between patient and tumour characteristics in linked patients with sarcoma when compared with those in the broader ACCORD dataset, and that patients with sarcoma with and without linkage to primary care were comparable (Supplementary Tables S1 and S2). Of the 441 linked patients, 377 patients were diagnosed with STS and 64 with BS. The sex distribution was similar in both groups, with males comprising 51% (*n* = 192) of patients with STS and 63% (*n* = 40) of patients with BS (*P* = 0.08). Patients with BS were on average younger, with a mean age of 42 years compared with 54 years for patients with STS. A higher proportion of patients with BS were diagnosed during AYA years (23% [*n* = 15] of patients with BS aged 15–25 years compared with 6% [*n* = 24] of patients with STS [*P*<0.001]) (Table 1).

**Table 1.** Baseline characteristics of patients with sarcoma with linked data

**Characteristics**	**Soft tissue sarcoma (*n* = 377)**	**Bone sarcoma (*n* = 64)**	***P*-value^a^**
Male sex	192 (51%)	40 (63%)	0.08

Age at diagnosis (years)			<0.001
15–25	24 (6%)	15 (23%)	
26–35	46 (12%)	12 (19%)	
36–45	54 (14%)	9 (14%)	
46–55	74 (20%)	12 (19%)	
56–65	60 (16%)	11 (17%)	
66–75	70 (19%)	1 (2%)	
≥76	49 (13%)	4 (6%)	
Mean (SD, range)	54 (18, 17–95)	42 (18, 15–83)	<0.001

Year of diagnosis			0.054
2002–2006	4 (1%)	2 (3%)	
2007–2011	70 (19%)	20 (31%)	
2012–2016	173 (46%)	25 (39%)	
2017–2021	130 (34%)	17 (27%)	

Lookback period^b^			0.10
<12 months	109 (29%)	25 (39%)	
12–24 months	268 (71%)	39 (61%)	
Median (IQR)	731 (307–731)	693 (152–731)	

GP clinical activity in 6 months pre-diagnosis			
GP visit	316 (84%)	55 (86%)	0.67
Imaging request (any)	134 (36%)	21 (33%)	0.67
Ultrasound request	101 (27%)	10 (16%)	0.06
X-ray request	35 (9%)	12 (19%)	0.02
CT request	52 (14%)	14 (22%)	0.09
MRI request	26 (7%)	3 (5%)	0.51
Bone density request	7 (2%)	5 (8%)	0.007
Prescription	163 (43%)	27 (42%)	0.88
Blood request	103 (27%)	14 (22%)	0.36

a P*-value from* χ*^2^ test.*

b *Time from being active in a linked general practice to diagnosis (maximum 2 years). CT = computed tomography. IQR = interquartile range. MRI = magnetic resonance imaging. SD = standard deviation.*

### Tumour characteristics

High-grade disease was common in both groups, comprising 70% of BS (*n* = 28) and 47% of STS (*n* = 104) diagnoses, where reported. Late-stage diagnosis (stage 3 and 4) was more prevalent among patients with STS (59% [*n* = 123] of patients with STS versus 22% [*n* = 8] of patients with BS, *P*<0.001). A considerable proportion of tumours in both groups were ≥5 cm in diameter at diagnosis (59% [*n* = 198] of STS and 54% [*n* = 27] of BS), with STSs being larger on average than BSs at diagnosis (mean 7.4 cm, range 0.3–34.5 in STS versus mean 5.3 cm, range 0.6–14.6 in BS, *P* = 0.009) (Table 2).

**Table 2.** Baseline characteristics of patients with sarcoma at first consult in specialist centre

**Characteristics**	**Soft tissue sarcoma (*n* = 377)**	**Bone sarcoma (*n* = 64)**	***P*-value^a^**
Tumour location			
Extremities	249 (66%)	—	—
Centralised^b^	96 (25%)	—	—
Both	27 (7%)	—	—
Missing	5 (1%)	—	—

Tumour behaviour			0.26
Malignant	212 (56%)	43 (67%)	
Intermediate	75 (20%)	10 (16%)	
Benign	76 (20%)	9 (14%)	
Missing	14 (4%)	2 (3%)	

Pre-diagnosis biopsy			0.38
Yes	34 (9%)	3 (5%)	
No	235 (62%)	36 (56%)	
Missing	108 (29%)	25 (39%)	

Grade			0.008
High	104 (28%)	28 (44%)	
Low	116 (31%)	12 (19%)	
Missing	157 (42%)	24 (38%)	

Stage			<0.001
Early (stage I or II)	85 (23%)	28 (44%)	
Late (stage III or IV)	123 (33%)	8 (13%)	
Missing	169 (45%)	28 (44%)	

Depth			0.006
Deep	220 (58%)	64 (100%)	
Superficial	39 (10%)	0 (0%)	
Missing	118 (31%)	0 (0%)	

≥5 cm diameter			0.54
Yes	198 (53%)	27 (42%)	
No	140 (37%)	23 (36%)	
Missing	39 (10%)	14 (22%)	

Largest dimension (cm)			
Mean (SD, range)	7.4 (6.1, 0.3–34.5)	5.3 (35.1, 0.6–14.6)	0.009

Symptom present at first specialist consult			
Mass			<0.001
Yes	258 (68%)	22 (34%)	
No	95 (25%)	34 (53%)	
Missing	24 (6%)	8 (13%)	
Pain			<0.001
Yes	137 (36%)	45 (70%)	
No	194 (51%)	10 (16%)	
Missing	46 (12%)	9 (14%)	
Systemic symptoms			0.55
Yes	51 (14%)	7 (11%)	
No	282 (75%)	50 (78%)	
Missing	44 (12%)	7 (11%)	

Referred by GP			0.64
Yes	206 (55%)	31 (48%)	
No	115 (31%)	20 (31%)	
Missing	56 (15%)	13 (20%)	

a P*-value from* χ*^2^ test.*

b *Centralised tumour location is defined as soft tissue sarcomas of intra-abdominal, intrapelvic, intrathoracic, mediastinal, retroperitoneal, or gynaecological origin. SD = standard deviation.*

### Presentation and referral

Patients with STS were more likely than patients with BS to present with a painless mass (68% [*n* = 258] versus 34% [*n* = 22] *P*<0.001), while patients with BS were more likely to present with pain (70% [*n* = 45] versus 36% [*n* = 137], *P*<0.001). Approximately half of patients with sarcoma in both groups were referred to a specialist by their GP (55% [*n* = 206] of STS and 48% [*n* = 31] of BS), with about 1 in 10 having a non-diagnostic biopsy (9% [*n* = 34] of STS and 5% [*n* = 3] of BS) (Table 2).

### Primary care utilisation before diagnosis

Among the study cohort, regarding who had ≥1 encounter in a linked general practice in the year pre-diagnosis, approximately four in five patients with sarcoma visited a GP in the 6 months leading up to diagnosis (84% [*n* = 316] of STS and 86% [*n* = 55] of BS) (Table 1). Repeat GP visits were common, with half of patients with STS (52%; *n* = 197) and BS (47%; *n* = 30) visiting their GP ≥4 times in the 6 months pre-diagnosis (Table 3). Patients had a median of 3–4 GP visits in the 6 months pre-diagnosis (Table 3) and six GP visits in the year pre-diagnosis (Supplementary Table S3). Pre-diagnostic imaging was ordered by GPs for around one-third of patients (33% [*n* = 21] of BS and 36% [*n* = 134] of STS) in the 6 months before diagnosis (Table 1). Patients with STS were most likely to be referred for an USS (27% [*n* = 101] versus 16% [*n* = 10], *P* = 0.06), and patients with BS were more likely to be referred for an X-ray (19% [*n* = 12] versus 9% [*n* = 35], *P* = 0.02) (Table 1). In the 6 months pre-diagnosis, multiple imaging (≥2 scans) was requested in 21% (*n* = 80) of patients with STS and 19% (*n* = 12) of patients with BS (Table 3). Repeat USS were requested in 5% (*n* = 3) of patients with BS and 7% (*n* = 27) of patients with STS and repeat CT and DXA scan were requested in 5% (*n* = 3) and 8% (*n* = 5) of patients with BS, respectively (Table 3).

**Table 3.** Number of GP clinical events in the 6 months before sarcoma diagnosis among the study cohort of 441 patients with sarcoma who had ≥1 linked GP encounter in the year pre-diagnosis

**Type of clinical activity^a^**	**Soft tissue sarcoma (*n* = 377)**	**Bone sarcoma (*n* = 64)**	***P*-value^b^**
GP visits			0.76
0	61 (16%)	9 (14%)	
1–3	119 (32%)	25 (39%)	
4–6	82 (22%)	11 (17%)	
7–9	60 (16%)	11 (17%)	
≥10	55 (15%)	8 (13%)	
Mean (SD, range)	5 (5.7, 0–34)	5 (4.4, 0–19)	
Median (IQR)	4 (1–7)	3 (1–7)	

Imaging requests (any type)			0.045
0	243 (64%)	43 (67%)	
1	54 (14%)	9 (14%)	
2	42 (11%)	1 (2%)	
3	28 (7%)	6 (9%)	
≥4	10 (3%)	5 (8%)	
Mean (SD, range)	1 (1.1, 0–6)	1 (1.4, 0–5)	
Median (IQR)	0 (0–1)	0 (0–1)	

Prescriptions			0.32
0	214 (57%)	37 (58%)	
1	80 (21%)	11 (17%)	
2	29 (8%)	4 (6%)	
3	28 (7%)	3 (5%)	
≥4	26 (7%)	9 (14%)	
Mean (SD, range)	1 (1.6, 0–9)	1 (2.0, 0–9)	
Median (IQR)	0 (0–1)	0 (0–2)	

Blood requests			0.47
0	274 (73%)	50 (78%)	
1	64 (17%)	11 (17%)	
2	24 (6%)	1 (2%)	
3	9 (2%)	2 (3%)	
≥4	6 (2%)	0 (0%)	
Mean (SD, range)	0 (0.9, 0–5)	0 (0.7, 0–3)	
Median (IQR)	0 (0–1)	0 (0–0)	

Ultrasound requests			0.16
0	276 (73%)	54 (84%)	
1	74 (20%)	7 (11%)	
≥2	27 (7%)	3 (5%)	

X-ray requests			0.03
0	342 (91%)	52 (81%)	
1	32 (8%)	12 (19%)	
≥2	3 (1%)	0 (0%)	

CT scan requests			0.20
0	325 (86%)	50 (78%)	
1	44 (12%)	11 (17%)	
≥2	8 (2%)	3 (5%)	

MRI scan requests			0.73
0	351 (93%)	61 (95%)	
1	24 (6%)	3 (5%)	
≥2	2 (1%)	0 (0%)	

Number of bone density requests in 6 months pre-diagnosis			0.007
0	370 (98%)	59 (92%)	
1	7 (2%)	5 (8%)	

a *Maximum one event counted per day.*

b P*-value from* χ*^2^ test. CT = computed tomography. IQR = interquartile range. MRI = magnetic resonance imaging. SD = standard deviation.*

### Trends in primary care utilisation

**Figure 1.**
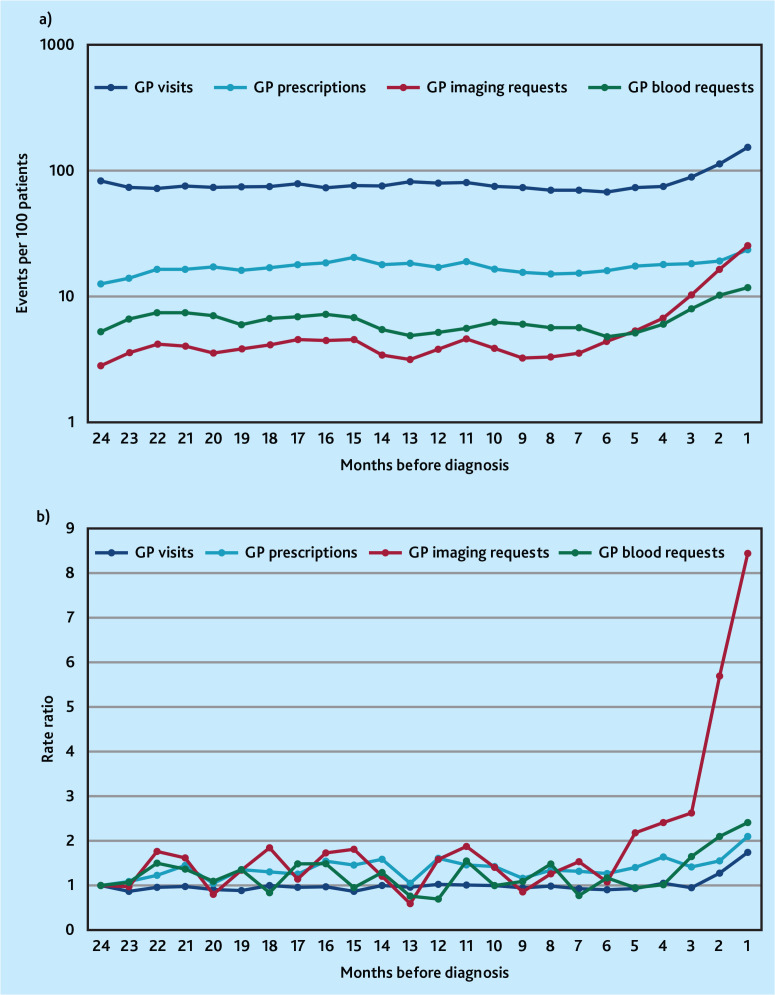
Monthly rate and rate ratio of different types of GP clinical activity in the 24 months before sarcoma diagnosis. 1a) shows incident rates (3-month moving average, log scale). 1b) shows rate ratios compared with baseline rate at 24 months pre-diagnosis.

The monthly rate of GP imaging requests progressively increased from 6 months before sarcoma diagnosis (the statistically determined inflection point and estimated diagnostic window), peaking at eight times the baseline rate immediately before diagnosis (IRR 8.43, 95% confidence interval [CI] = 3.92 to 18.15, *P*<0.001) (Figure 1, Supplementary Tables S5 and S6). This increase was initially gradual, followed by a more rapid rise in the 3 months preceding cancer diagnosis. This trend was accompanied by much smaller increases in the rate of GP visits (from −3 months), prescriptions (−2 months), and blood test requests (−4 months). Comparison of rates during the periods pre- (−24 to −7 months) and post- (−6 to 0 months) the diagnostic window inflection point found a three-fold increase in imaging requests (IRR 2.87, 95% CI = 2.39 to 3.46) with only marginal increases in all other events (IRRs <1.4) (Figure 1, Supplementary Tables S5 and S6).

**Figure 2.**
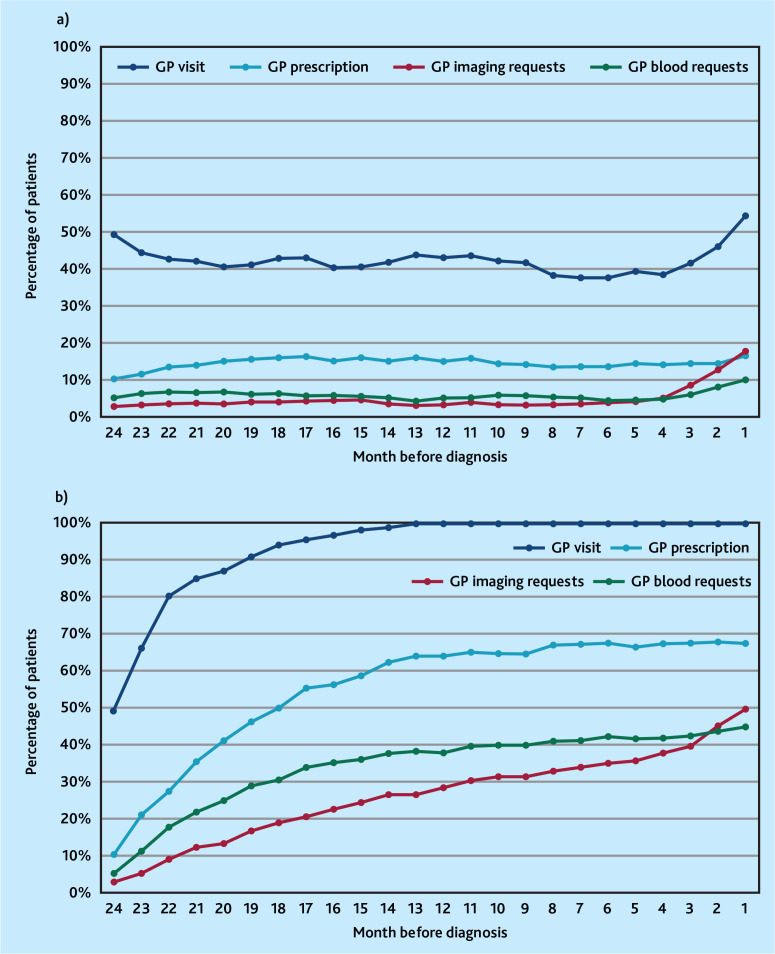
Monthly proportion of patients with sarcoma receiving different types of GP clinical activity in the 24 months before diagnosis. 2a) shows monthly incident percentage using 3-month moving average. 2b) shows cumulative percentage from 24 months pre-diagnosis.

On examining the proportion of patients with sarcoma experiencing GP events over time, the monthly percentage of patients with a GP imaging request progressively increased from 4 months pre-diagnosis, from a baseline of 3% up to 18% in the month immediately preceding sarcoma diagnosis (Figure 2, Supplementary Table S7). The monthly proportion of patients with a GP visit increased steadily over the same period from a baseline of around 40% of patients to 55% in the month pre-diagnosis. The proportion of patients receiving prescriptions or blood test requests remained stable over the 24 months pre-diagnosis (Figure 2, Supplementary Table S7).

## Discussion

### Summary

To the authors’ knowledge, this is the first study that has examined general practice healthcare utilisation over time before a sarcoma diagnosis. Patients with as-yet-undetected sarcoma, who visited a GP in the year pre-diagnosis, experience increased clinical activity in primary care from 6 months before diagnosis, predominantly in the form of imaging requests. This has indicated that many patients with sarcoma present to their GP several months pre-diagnosis with symptoms prompting imaging investigation. During this 6-month period, patients with sarcoma on average visited their GP 3–4 times, one-third were referred for imaging (predominantly USS and X-rays), and one in five had multiple scans requested. This period represents a ‘diagnostic window’, where potential opportunities exist for expediting sarcoma diagnosis in some patients, if supported by interventions to help identify these patients, optimise their investigation, and overcome barriers to timely diagnosis.

### Strengths and limitations

Using linked primary care data is an important strength of this study as almost all patients with sarcoma first present in this setting.^8^^,^^16^ Using two general practice datasets, with broad coverage of Victoria and a representative sample of the Australian population, enabled a sample size large enough to longitudinally examine several GP clinical events. Statistical estimation of inflection points increased reliability of findings.^24^ Data on GP encounters, prescriptions, and investigations are automatically recorded, and time stamped, increasing accuracy and completeness. The ACCORD dataset captures the majority of sarcoma in Victoria and ensured access to accurate tumour-related data.

Some information was not available, including indications for investigations, imaging results, and referral details. Future research into these factors and reasons for consultations could help understand causes of diagnostic delays. Some tumour characteristics were partially missing, but data on the primary outcomes (diagnosis date and tumour site) were complete. The small increases in GP events (apart from imaging) when comparing periods pre- and post- the estimated diagnostic window inflection point could reflect regression to the mean.

In Australia, patients are not restricted to visiting a single general practice and can be simultaneously registered with multiple practices, even if they have not consulted there for several years. To ensure only data from patients ‘actively’ registered at a GP practice were included, any patients who had not attended a linked practice in the year pre-diagnosis were excluded. This could introduce selection bias, as these patients may have higher levels of primary healthcare utilisation than those who did not see a GP in this period. However, the effects of this are likely to be minimal as studies have shown that almost all patients with sarcoma (87%–90%) will present in primary care^8^^,^^16^ in the 12 months before diagnosis (median time of first GP presentation = 65 days before diagnosis, range 42–133 days).^8^ Additionally, sensitivity analyses showed that the study cohort were similar to all patients with sarcoma in the broader ACCORD dataset across a range of patient and tumour characteristics. The results are therefore likely to be generalisable to many patients with sarcoma who will see a GP at least once in the year pre-diagnosis. If all patients with sarcoma who had attended a linked general practice were included, many ‘inactive’ patients who were receiving primary care elsewhere would be captured, which would underestimate primary care activity pre-diagnosis. Owing to the structure of Australian primary care, patient activity occurring at general practices outside the datasets will not be captured; however, this is likely to be minimal in the ‘actively’ registered sarcoma cohort, as 90% of Australians visit a regular general practice.^25^

### Comparison with existing literature

European studies have reported average diagnostic intervals^26^ (time from first clinical presentation to sarcoma diagnosis) ranging from 2–6 months.^7^^–^^10^^,^^16^ These studies involved <200 patients, used retrospectively collected survey data, and showed wide variation between countries. The present study estimates the diagnostic window length in 441 Australian patients with sarcoma. Rather than a single estimate of average time to diagnosis, this measure collates data from multiple pre-diagnostic events in a population of patients with sarcoma to identify when clinical activity first starts to increase.^24^^,^^27^^–^^29^ This inflection point indicates the start of the ‘diagnostic window’, within which potential opportunities exist for expediting diagnosis in some patients, with longer diagnostic windows signalling that earlier action could potentially be taken. The diagnostic window therefore represents the maximum time that diagnosis could potentially be brought forward in some patients, if supported by diagnostic advances to help identify them. Consistent with previous diagnostic interval estimates, the present study found a diagnostic window of up to 6 months before sarcoma diagnosis, where there are increased GP visits and imaging requests, and potential opportunities for earlier diagnosis in some patients.

UK studies of GP consultations before sarcoma diagnosis found that 41%–50% of patients with BS and 25%–32% of patients with STS had ≥3 GP consultations pre-referral and 15% and 10%, respectively, had ≥5 consultations.^12^^,^^17^ In both studies, consultations could occur at any point from first relevant GP presentation. The authors are not aware of previous evidence examining the timing of GP consultations pre-diagnosis, or using Australian data. The present study has shown that around half of patients with sarcoma have ≥4 GP visits in the 6 months pre-diagnosis, suggesting more potential opportunities to expedite diagnosis exist and in a larger proportion of patients than previously described. Furthermore, the study found that increases in GP visits and imaging requests were concentrated in the 3–6 months before sarcoma diagnosis.

Regarding pre-diagnostic imaging, a Swedish study found 64% of patients with sarcoma had imaging requested at their first medical presentation (to primary care or emergency department).^30^ An Australian study of 21 clinicians and 22 patients with sarcoma reported prolonged intervals in after-test referrals, with some patients having several scans pre-diagnosis.^5^ The current authors’ study builds on these findings by longitudinally examining GP requests for five imaging modalities, showing that GP imaging requests increase 6 months before sarcoma diagnosis, and in this period one-third of patients with sarcoma have imaging and many experience multiple or repeat scans. Unlike in many other healthcare settings, there are not long waiting times for imaging or specialist care in the Australian system. Potential causes of delay may therefore exist after patients undergo imaging, including owing to false negative results from using modalities with poor diagnostic accuracy for sarcoma or from onward referrals to non-sarcoma specialists.

### Implications for research and practice

**Figure 3.**
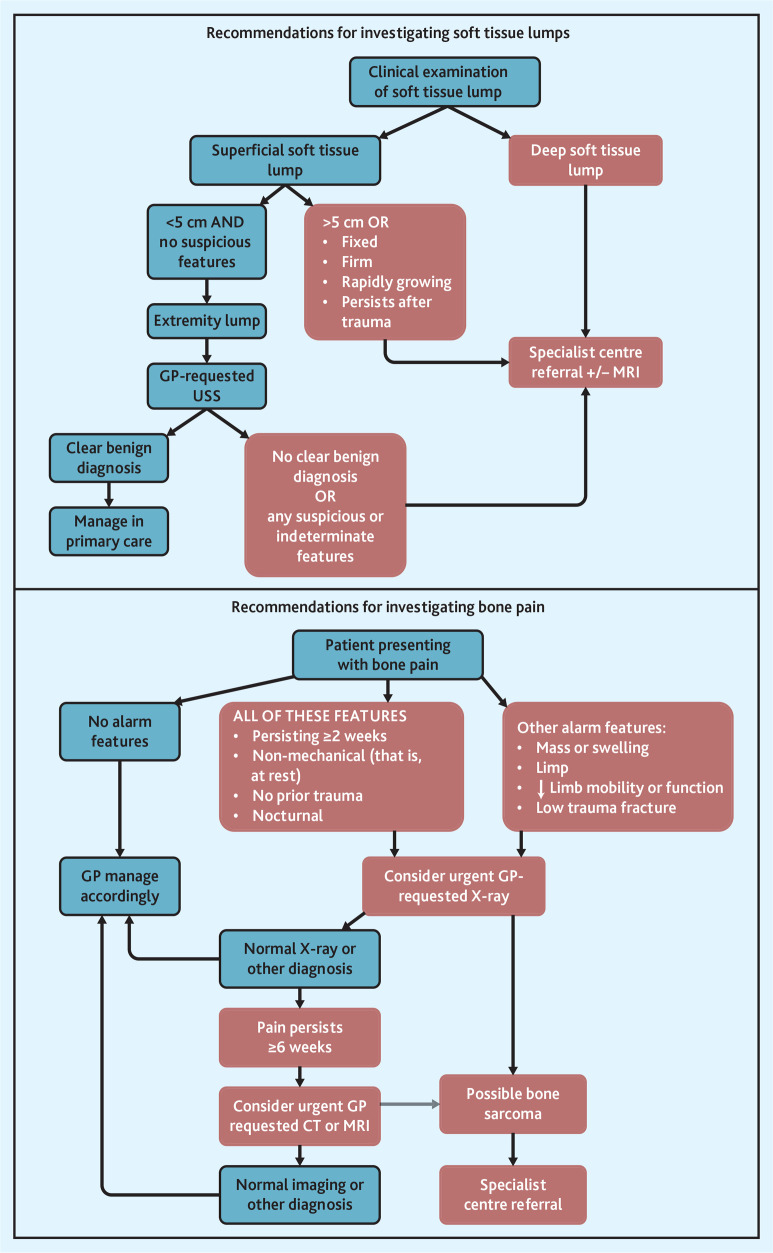
Summary of recommendations for investigation of bone pain and soft tissue lumps in primary care. Blue boxes represent primary care management; red boxes represent specialist centre management; clear benign diagnosis refers to a confirmed reported diagnosis of a benign nature, for example, lipoma, with no additional suspicious or uncertain features. CT = computed tomography. MRI = magnetic resonance imaging. USS = ultrasound scan.

A window of opportunity exists where the diagnosis of sarcoma could potentially be accelerated in primary care by several months in some patients if supported by targeted interventions and diagnostic advancements. The types of GP diagnostic activity that occur early in this window to inform development of future interventions have been identified, which could, in turn, improve the timeliness of diagnosis. During this window, many patients with sarcoma report multiple GP visits and wide variability exists in clinical practice, for example, one-third have GP imaging, different imaging modalities are used, and many have multiple scans. Improving awareness, scope, and consistency of guidelines to optimise investigation of bone pain and soft tissue lumps could improve timely diagnosis. Australia’s sarcoma optimal care pathway (OCP)^31^ recommends urgent X-ray in persistent, non-mechanical bone pain in the absence of prior trauma, lasting >6 weeks; however, only one in five patients with BS in this study had a GP X-ray request despite bone pain being the most common presenting feature. The OCP also recommends that all soft tissue lumps that are deep, growing, >5 cm, or not caused by trauma should be directly referred to a specialist for gold-standard MRI imaging. However, no guidance is given on the management of lumps outside these criteria. Other international guidelines (National Institute for Health and Care Excellence [NICE] and European Society of Musculoskeletal Radiology [ESSR]) recommend triage imaging with ultrasound to identify patients warranting specialist referral,^32^^,^^33^ with CT having a limited role, except for intrathoracic or intra-abdominal lesions or where MRI is contraindicated.^32^^,^^34^ Clear, high-quality reporting and avoiding downstream delays after abnormal imaging in BS and STS are also essential, as GP imaging requests increased up to 6 months before sarcoma diagnosis. Only half of patients with sarcoma were directly referred by their GP to specialised centres (recommended for optimal sarcoma outcomes),^35^^,^^36^ revealing opportunities for strategic interventions in patients with possible BS or STS to increase direct GP- to-specialist centre referrals and redirect referrals from other entry points. Based on these findings, a summary guide has been provided by the authors to optimise investigation of bone pain and soft tissue lumps in primary care to support earlier diagnosis and improve patient outcomes (Figure 3).

## Data

The data that support the findings of this study are available from the VCCC Alliance Data Connect. Restrictions apply to the availability of these data, which were used under approvals for this study. Data are available from https://vcccalliance.org.au/our-work/research-and-translation/data-connect/how-to-access-the-hub/with the permission of BioGrid.
